# Squirting Cucumber, *Ecballium elaterium* (L.) A. Ritch: An Update of Its Chemical and Pharmacological Profile

**DOI:** 10.3390/molecules29184377

**Published:** 2024-09-14

**Authors:** Attilio Anzano, Bruna de Falco, Laura Grauso, Virginia Lanzotti

**Affiliations:** Department of Agricultural Sciences, Università di Napoli Federico II, Via Università 100, 80055 Portici, NA, Italy; attilio.anzano@unina.it (A.A.); bruna.defalco@unina.it (B.d.F.); laura.grauso@unina.it (L.G.)

**Keywords:** cucurbitacins, organic compounds, bioactive metabolites, methods of analysis, biological activity

## Abstract

*Ecballium elaterium*, also known as squirting cucumber, is a plant which is widespread in temperate regions of Europe, Africa and Asia. The plant is considered to be one of the oldest used drugs. In the last decades, *E. elaterium* has been widely studied as a source of triterpene metabolites named cucurbitacins, often found as glycosylated derivatives, used by the plant as defensive agents. Such metabolites exhibit several biological activities, including cytotoxic, anti-inflammatory, and anti-cancer. Interestingly, the bioactive properties of *E. elaterium* extracts have been investigated in dozens of studies, especially by testing the apolar fractions, including the essential oils, extracted from leaves and fruits. The purpose of this review is to provide an overview of the chemical profile of different parts of the plants (leaves, flowers, and seeds) analyzing the methods used for structure elucidation and identification of single metabolites. The pharmacological studies on the isolated compounds are also reported, to highlight their potential as good candidates for drug discovery.

## 1. Introduction

**Figure 1:**
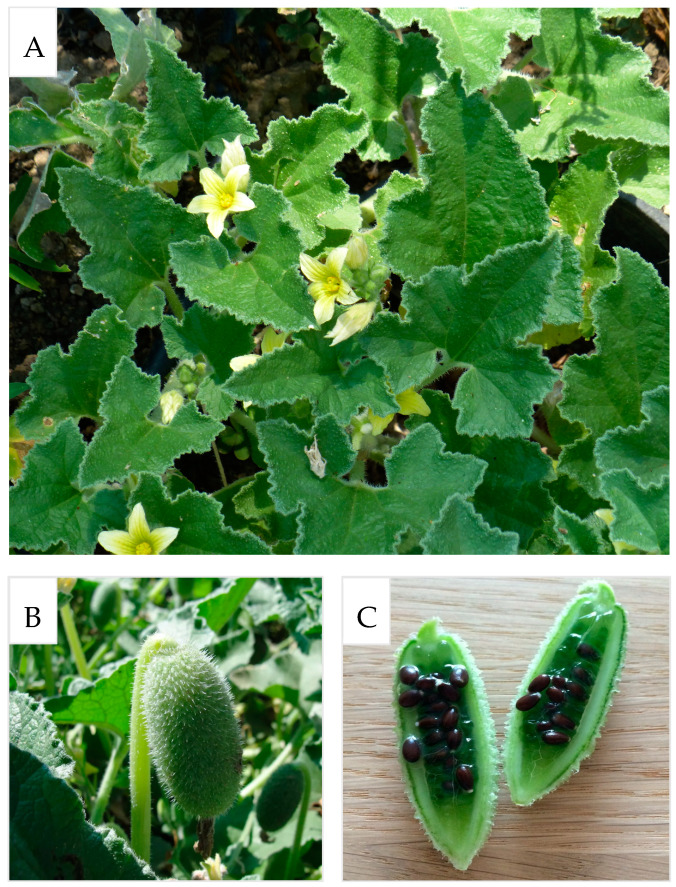
*Ecballium elaterium* leaves and flowers (**A**), fruits (**B**), and seeds (**C**).

*Ecballium elaterium* (L.) A. Rich., is a herbaceous plant belonging to the *Cucurbitaceae* family that grows in the Mediterranean area, and which is the only one belonging to the *Ecballium* genus. It is commonly named squirting cucumber or exploding cucumber, for the ability of the fruit to violently disperse the seeds (from the Greek, *εκβαλλω* = to expel, to cast out). When the fruits reach a certain degree of ripening, or when an animal grabs the fruits and separates them from the stem, the internal pressure (up to 27 atm) throws the seeds away from the plant, allowing the plant to rapidly colonize wide areas. *E. elaterium* is native to Mediterranean areas, but it can be also found in northern Africa countries and temperate regions of Western Asia. It is considered an invasive species, as it can easily grow in rich soils and sunny environments, but it can grow in poor and drained soils too; it does not need much water, nor a specific pH range. Very recently Motti et al. (2023) documented the presence of this plant in the Ansanto Valley (Avellino province, Southern Italy) [1]. This finding is very interesting, since that place, named Mefite, can be considered a peculiar ecological niche, due to boiling mud lakes and vents with very high levels of natural CO_2_ emissions [1]. The plant grows as a perennial herb with wide, hairy, dark green and rough leaves, hairy and green stems that can grow up to 0.3 m, green, oval and hairy fruits, and pale yellow flowers that flourish between June and August (Figure 1) [2,3]. The small, dark brown, round seeds can be found inside the fruit, together with the fruit juice, which is the main part known for both beneficial and harmful properties. In traditional medicine, the dried fruit juice, called “elaterium”, has been used to treat rheumatism, jaundice, sinusitis, fever, liver disorders, and constipation, especially in medicine in Tunisia, Algeria and Turkey, and today it is still used in some Mediterranean medicinal sistems [1,4,5,6,7]. Recent studies on the plant fruits, seeds and roots extracts confirmed these biological activities. The beneficial properties of the fruit juice have been widely studied, and are attributed to the cucurbitacins, a class of tetracyclic triterpenes mainly found in *Cucurbitaceae*, but also to other bioactive components of the plant [8].

Several cucurbitacins have been isolated over the last years, ten of which have been identified in *E. elaterium.* Cucurbitacins can also be found as glycosides with different monosaccharides, mainly d-glucose and l-rhamnose, but these molecules are much less studied, and little is known about their effects on living organisms [9]. The fruit and the fruit juice are also known to be toxic for humans and animals if eaten, because of the high quantity of active principles contained in the juice, which can lead to severe damage, such as accelerated pulse, nervousness, dyspnea, anorexia, diarrhea, and, in some extreme cases, death by convulsions and asphyxia [9]. Salhab provided a list of medical cases in which exposure of people to the *E. elaterium* juice led to edema at a nasal, pharynx or uvular level, throat soreness, shortness of breath, conjunctivitis, and cardiac and renal failure [10]. These consequences are caused mainly by the high content of cucurbitacins, which are heavy purgatives and have a strong bitter taste. The first cucurbitacin was extracted from *E. elaterium*, attaching importance to this plant that is not edible and which is toxic, and thus it was less important than the other edible *Cucurbitaceae*. In fact, the concentration of cucurbitacin in *E. elaterium*, around 3.84% *w*/*w* (weight per weight) in the fruits, 1.34% in the stems and 0.34% in the leaves, ref. [11] is considerably higher than in other edible *Cucurbitaceae*, where the cucurbitacin content is usually between 0.1 and 0.3% and, anyway, below 1% [9,12]. Because of this, *E. elaterium* is considered a good source of cucurbitacins, and in some cases it has also been cultured in vitro with the aim of producing cucurbitacins [9]. This, together with the many different biological activity possessed by different parts of the plant, make this plant quite important from the chemical and pharmacological point of view.

Recently, Ielciu et al. wrote a review about *E. elaterium* and *Bryonia alba* highlighting the main characteristics of these two plants belonging to the *Cucurbitaceae* family [13]. Other than this study, no other recent review works can be found specifically about *E. elaterium*. The scientific literature regarding this plant is relatively poor, especially concerning the chemical composition of the different parts of the plant. The purpose of this review work is to provide an overview of the chemical profile of *E. elaterium* leaves, seeds and fruits in order to give an overview of all the molecules that have been identified up to now. The biological activities reported for the different plant extracts are also reviewed, as well as the pharmacological studies on the isolated cucurbitacins, to highlight their potential as good candidates for drug discovery.

## 2. Chemical Composition of the Plant Leaves, Fruits and Seeds

The different parts of the plant have been studied to determine their chemical composition and to find the role of such metabolites in the plant. Table 1 lists all the metabolites extracted and characterized to date, based on the very different chemical structure belonging to the main classes of fatty acids, carbohydrates, alkanes, esters, aldehydes, tocopherols, terpenes and their derivatives, flavonoids, and phytohormones.

Comparison of the chemical profiles evidenced differences in the chemical composition of the different parts of the plant. In particular, terpenes and volatiles were reported from leaves, along with flavonoids, phenolics and sugars. Triterpenoids, based on the oleanoic acid structure, were detecetd in the plant fruits. Unsaturated fatty acids and steroids were reported in seeds.

**Table 1:** Chemical composition of *Ecballium elaterium* leaves, fruits and seeds.

Compounds	Formula	Chemical Structure	Quantity			Ref.
			Leaves	Fruits	Seeds	
Benzaldehyde (**1**)	C_7_H_6_O	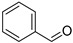	12.3% of total area ^1,2^	-	-	[2]
Benzeneacetaldehyde (**2**)	C_8_H_8_O	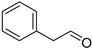	0.9% of total area ^1,2^	-	-	[2]
β-Bisabolol (**3**)	C_15_H_26_O	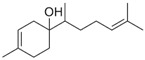	0.7% of total area ^1,2^	-	-	[2]
Butyl cyclohexyl phthalate (**4**)	C_18_H_24_O_4_	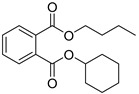	1.3% of total area ^1,2^	-	-	[2]
Cubitene (**5**)	C_20_H_32_	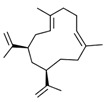	1.2% of total area ^1,2^	-	-	[2]
β-Cyclocitral (**6**)	C_10_H_16_O	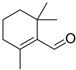	0.8% of total area ^1,2^	-	-	[2]
β-Damascone (**7**)	C_13_H_20_O	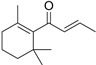	1.1% of total area ^1,2^	-	-	[2]
n-Decanal (**8**)	C_10_H_20_O	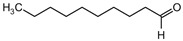	1.5% of total area ^1,2^	-	-	[2]
4,6-Dimethyl-3,5,7- trioxatetracyclo [7.2.1.0(4,11).0(6,10)] dodecane (**9**)	C_11_H_16_O_4_	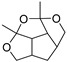	1.1% of total area ^1,2^	-	-	[2]
6-Ethyl-3-octyl isobutylester phthalic acid (**10**)	C_22_H_34_O_4_	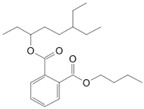	0.8% of total area ^1,2^	-	-	[2]
Ethylsorbate (**11**)	C_8_H_12_O_2_	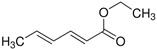	0.8% of total area ^1,2^	-	-	[2]
cis-Eudesma,6,11 diene (**12**)	C_15_H_24_	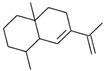	0.9% of total area ^1,2^	-	-	[2]
Eudesmol (**13**)	C_15_H_26_O	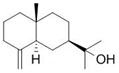	1.0% of total area ^1,2^	-	-	[2]
10-epi-γ-Eudesmol (**14**)	C_15_H_26_O	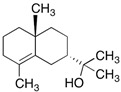	2.1% of total area ^1,2^	-	-	[2]
α-Fenchocamphorone (**15**)	C_9_H_14_O	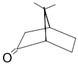	2.0% of total area ^1,2^	-	-	[2]
Germacrene A (**16**)	C_15_H_24_	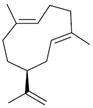	0.7% of total area ^1,2^	-	-	[2]
Hexadecanoicacid, methyl ester (**17**)	C_17_H_34_O_2_	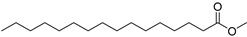	2.8% of total area ^1,2^	-	-	[2]
Hinesol (**18**)	C_15_H_26_O	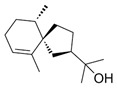	17.2% of total area ^1,2^	-	-	[2]
(*E*)-β-Ionone (**19**)	C_13_H_20_O	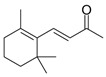	7.8% of total area ^1,2^	-	-	[2]
2-Isobutylthiazole (**20**)	C_7_H_11_NS	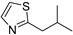	1.6% of total area ^1,2^	-	-	[2]
Isolongifolene (**21**)	C_15_H_24_	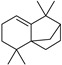	1.9% of total area ^1,2^	-	-	[2]
epi-Laurenene (**22**)	C_20_H_32_	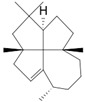	1.3% of total area ^1,2^	-	-	[2]
Longifolol (**23**)	C_15_H_26_O	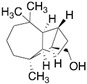	0.7% of total area ^1,2^	-	-	[2]
2-Methyl-7-octadecyne (**24**)	C_19_H_36_	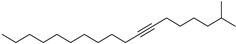	2.6% of total area ^1,2^	-	-	[2]
E-Nerolidol (**25**)	C_15_H_26_O	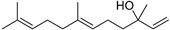	1.2% of total area ^1,2^	-	-	[2]
Neryl acetone (**26**)	C_13_H_22_O	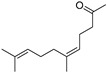	2.5% of total area ^1,2^	-	-	[2]
Nonadecane (**27**)	C_19_H_40_	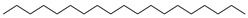	1.5% of total area ^1,2^, 0.9% of total area ^1^	1.4% of total area ^1^	-	[2]
Safranal (**28**)	C_10_H_14_O	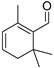	1.4% of total area ^1,2^	-	-	[2]
*o*-Tolualdehyde (**29**)	C_8_H_8_O	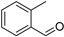	0.8% of total area ^1,2^	-	-	[2]
Vestitenone (**30**)	C_12_H_18_O	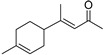	1.4% of total area ^1,2^	-	-	[2]
p-Vinylguaiacol (**31**)	C_9_H_10_O_2_	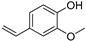	2.1% of total area ^1,2^	-	-	[2]
(24*S*)-Ethyl-5α-cholesta-7,22,25-trien-3β-ol (**32**)		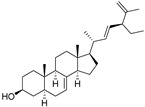	0.4mg/g of dry leaves	-	-	[14]
1-Allyl-1-but-3-enyl-1-silacyclobutane (**33**)	C_10_H_18_Si	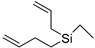	1.51% of total area ^2^	-	-	[15]
Carvacrol (**34**)	C_10_H_14_O	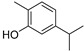	6.09% of total area ^2^	-	-	[15]
3,4-Dimethylheptane (**35**)	C_9_H_20_	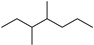	3.25% of total area ^2^	-	-	[15]
(*E*)-5-Eicosene (**36**)	C_20_H_40_	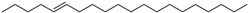	6.51% of total area ^2^	-	-	[15]
n-Hentriacontane (**37**)	C_31_H_64_	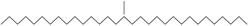	73,97% of total area ^2^	-	-	[15]
Limonene dioxide (**38**)	C_10_H_16_O_2_	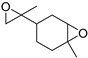	7.61% of total area ^2^	-	-	[15]
Linolenic acid methyl ester (**39**)	C_19_H_32_O_2_	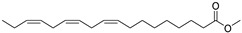	1.04% of total area ^2^	-	-	[15]
Loliolide (**40**)	C_11_H_16_O_3_	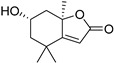	1.66% of total area ^2^	-	-	[15]
Neophytadiene (**41**)	C_20_H_38_	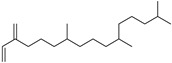	4.89% of total area (Hexane extract) ^2^	-	-	[15]
n-Pentacosane (**42**)	C_25_H_52_	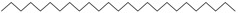	1.75% of total area ^2^	-	-	[15]
7,10-Pentadecadiynoic acid (**43**)	C_15_H_22_O_2_	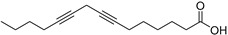	4.00% of total area ^2^	-	-	[15]
Phytol (**44**)	C_20_H_40_O	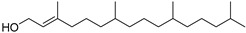	3.59% of total area ^2^	-	-	[15]
Propylhexedrine (**45**)	C_10_H_21_N	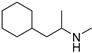	1.29% of total area ^2^	-	-	[15]
l-2-Tetramethyhexadecen-1-ol 3,7,11,15- (**46**)	C_20_H_40_O	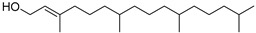	1.16% of total area ^2^	-	-	[15]
Thymol (**47**)	C_10_H_14_O	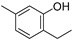	12.05% of total area ^2^	-	-	[15]
(*E*)-Anethol (**48**)	C_10_H_12_O	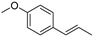	-	31.6% of total area ^1^	-	[16]
Dibuthylphtalate (**49**)	C_16_H_22_O_4_	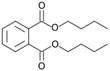	0.5% of total area ^1^	3.2% of total area ^1^	-	[16]
3-(6,6-Dimethyl-5-oxohept-2-enyl)-cyclohexanone (**50**)	C_15_H_24_O_2_	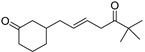	20.4% of total area ^1^	8.8% of total area ^1^	-	[16]
Eicosane (**51**)	C_20_H_42_	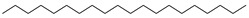	1.9% of total area ^1^	2.7% of total area ^1^	-	[16]
Estragol (**52**)	C_10_H_12_O	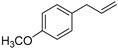	-	0.7% of total area ^1^	-	[16]
Henicosane (**53**)	C_21_H_44_	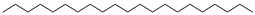	1.2% of total area ^1^	2.8% of total area ^1^	-	[16]
Heptadecane (**54**)	C_17_H_36_	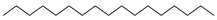	1.3% of total area ^1^	2.1% of total area ^1^	-	[16]
Hexadecane (**55**)	C_16_H_34_	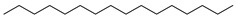	2.5% of total area ^1^	5.2% of total area ^1^	-	[16]
Hexahydrofarnesyl acetone (**56**)	C_18_H_36_O	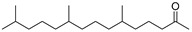	19.1% of total area ^1^	2.1% of total area ^1^	-	[16]
Isobutylphthalate (**57**)	C_12_H_14_O_4_	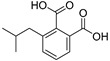	1.0% of total area ^1^	1.7% of total area ^1^	-	[16]
Limonene (**58**)	C_10_H_16_	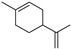	-	0.8% of total area ^1^	-	[16]
Methylheptadecane (**59**)	C_18_H_38_	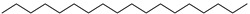	0.3% of total area ^1^	-	-	[16]
Nor-pristane (**60**)	C_18_H_38_	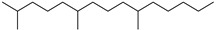	0.4% of total area ^1^		-	[16]
Octadecane (**61**)	C_18_H_38_	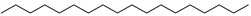	2.9% of total area ^1^	5.7% of total area ^1^	-	[16]
Octylhexanoate (**62**)	C_14_H_28_O_2_	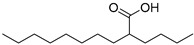	0.8% of total area ^1^	-	-	[16]
Octyloctanoate (**63**)	C_16_H_32_O_2_	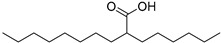	30.0% of total area ^1^	3.5% of total area ^1^	-	[16]
Pentadecane (**64**)	C_15_H_32_	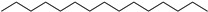	0.3% of total area ^1^	-	-	[16]
Phytene (**65**)	C_20_H_40_	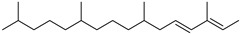	1.3% of total area ^1^	-	-	[16]
Pristane (**66**)	C_19_H_40_	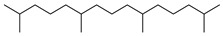	0.8% of total area ^1^	1.5 % of total area ^1^	-	[16]
p-Propylanisole (**67**)	C_10_H_14_O	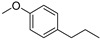	-	8.2% of total area ^1^	-	[16]
Tetracosane (**68**)	C_24_H_50_	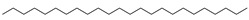	3.7% of total area ^1^	7.4% of total area ^1^	-	[16]
Tetradecane (**69**)	C_14_H_30_	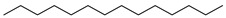	0.3% of total area ^1^	0.8% of total area ^1^	-	[16]
β-Thujone (**70**)	C_10_H_16_O	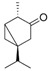	-	3.0% of total area ^1^	-	[16]
Tricosane (**71**)	C_23_H_48_	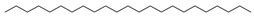	2.0% of total area ^1^	4.9% of total area ^1^	-	[16]
Caffeoylglucaric acid (**72**)	C_15_H_16_O_11_	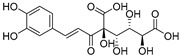	-	-	-	[17]
Catechin (**73**)	C_15_H_14_O_6_	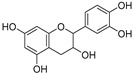	-	-	-	[17]
Catechin-3-*O*-rutinoside (**74**)	C_27_H_34_O_15_	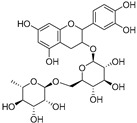	-	-	-	[17]
Colocynthoside B (**75**)	C_42_H_62_O_15_	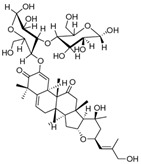	-	-	-	[17]
4-Feruloylquinic acid (**76**)	C_17_H_20_O_9_	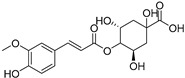	-	-	-	[17]
9-/ or 13-Hydroxy-9*Z*,11*E*-octadecadienoic acid (**77**)	C_18_H_32_O_3_	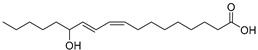	-	-	-	[17]
Linolenic acid (**78**)	C_18_H_30_O_2_	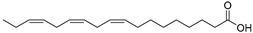	-	-	-	[17]
Procyanidin dimer (**79**)	C_30_H_26_O_12_	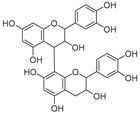	-	-	-	[17]
Shikimic acid hexoside isomer I (**80**)	C_13_H_20_O_10_	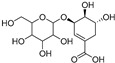	-	-	-	[17]
Shikimic acid hexoside isomer II (**81**)	C_13_H_20_O_10_	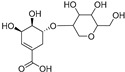	-	-	-	[17]
3,7,3′,4″-Tetrahydroxyflavanone (**82**)	C_15_H_12_O_6_	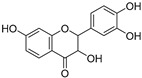	-	-	-	[17]
7,3′,4′-Trihydroxyflavanone (**83**)	C_15_H_10_O_5_	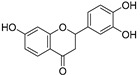	-	-	-	[17]
Trihydroxyflavanone-*O*-deoxyhexosyl-*O*-hexoside (**84**)	C_27_H_34_O_15_	n.d.	-	-	-	[17]
Trihydroxy-octadecadienoicacid (**85**)	C_18_H_32_O_5_	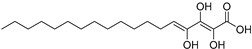	-	-	-	[17]
Trihydroxy-octadecenoicacid (**86**)	C_18_H_34_O_5_	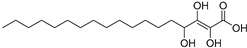	-	-	-	[17]
Elateroside A (**87**)	C_42_H_66_O_14_	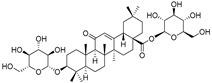	-	0.3 mg/kg fresh fruit	-	[18]
Elateroside B (**88**)	C_48_H_78_O_18_	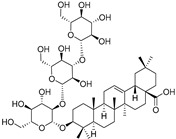	-	2 mg/kg fresh fruit	-	[18]
3-*O*-β-d-Glucopyranosyl-3β-hydroxyolean-12-en-28-oic acid 28-*O*-β-d-glucopyraonoside (**89**)	C_42_H_67_O_13_	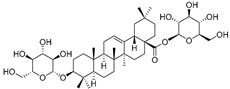	-	3 mg/kg fresh fruit	-	[18]
Oleanolic acid 3-*O*-β-d-glucopyranoside (**90**)	C_36_H_58_O_8_	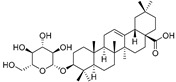	-	5 mg/kg fresh fruit	-	[18]
Oleanolic acid 3-*O*-β-d-glucopyranosyl-(1→2)-β-d-glucopyranoside (**91**)	C_42_H_68_O_13_	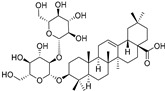	-	2 mg/kg fresh fruit	-	[18]
Arachidic acid (**92**)	C_20_H_40_O_2_	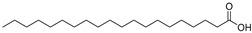	-	-	0.84% of total fatty acid	[19]
δ-5-Avenasterol (**93**)	C_29_H_48_O	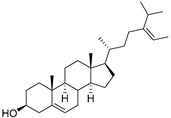	-	-	16.44 mg/100 g	[19]
δ-7-Avenasterol (**94**)	C_29_H_48_O	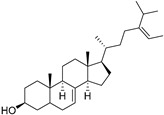	-	-	93.81 mg/100 g	[19]
Campesterol (**95**)	C_28_H_48_O	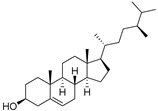	-	-	139.63 mg/100 g	[19]
δ-7-Campesterol (**96**)	C_28_H_48_O	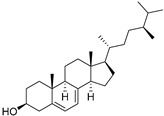	-	-	19.18 mg/100 g	[19]
Desmosterol (**97**)	C_27_H_44_O	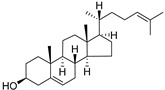	-	-	8.89 mg/100 g	[19]
Linoleic acid (**98**)	C_18_H_32_O_2_	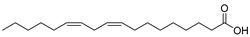	-	-	48.64% of total fatty acid	[19]
Myristic acid (**99**)	C_14_H_28_O_2_	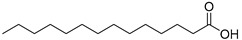	-	-	0.09% of total fatty acid	[19]
Oleic acid (**100**)	C_18_H_34_O_2_	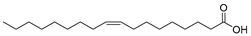	-	-	15.58% of total fatty acid	[19]
Palmitic acid (**101**)	C_16_H_32_O_2_	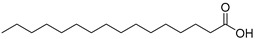	3.36% of total area^2^	-	4.09% of total fatty acid	[19]
Puninic acid (**102**)	C_18_H_30_O_2_	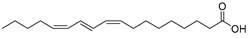	-	-	22.38% of total fatty acid	[19]
Sitostanol (**103**)	C_29_H_52_O	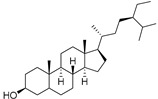	-	-	21.29 mg/100 g	[19]
β-Sitosterol (**104**)	C_29_H_50_O	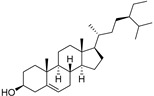	-	-	396.25 mg/100 g	[19]
Stearic acid (**105**)	C_18_H_36_O_2_	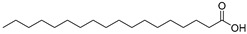	-	-	4.93% of total fatty acid	[19]
δ-7-Stigmastenol (**106**)	C_29_H_50_O	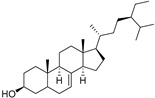	-	-	86.68 mg/100 g	[19]
Stigmasterol (**107**)	C_29_H_48_O	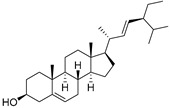	-	-	6.29 mg/100 g	[19]
α-Tocopherol (**108**)	C_29_H_50_O_2_	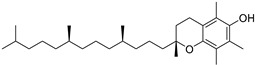	3.51% of total area ^2^	-	3.62 mg/100 g	[19]
β-Tocopherol (**109**)	C_28_H_48_O_2_	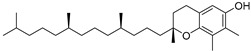	-	-	1.82 mg/100 g	[19]
γ-Tocopherol (**110**)	C_28_H_48_O_2_	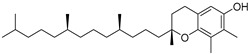	-	-	44.23 mg/100 g	[19]
δ-Tocopherol (**111**)	C_27_H_46_O_2_	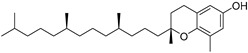	-	-	12.44 mg/100 g	[19]
Cycloeucalenol acetate (**112**)	C_32_H_52_O_2_	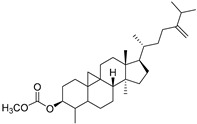	-	-	-	[20]
Fructose (**113**)	C_6_H_12_O_6_	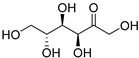	32% of total sugars	-	-	[21]
Glucose (**114**)	C_6_H_12_O_6_	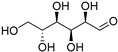	34% of total sugars	-	-	[21]
Inositol (**115**)	C_6_H_12_O_6_	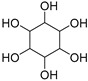	13% of total sugars	-	-	[21]
Raffinose (**116**)	C_18_H_32_O_16_	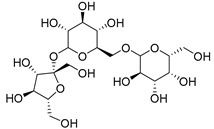	7% of total sugars	-	-	[21]
Sucrose (**117**)	C_12_H_22_O_11_	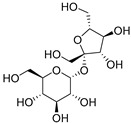	14% of total sugars	-	-	[21]
*N*-Ethyl-l-asparagine (**118**)	C_6_H_12_N_2_O_3_	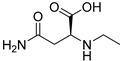	66.7mg/kg fresh areal parts	-	-	[22]
Phytomelin (**119**)	C_27_H_30_O_16_	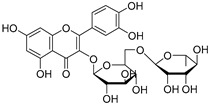	8.54 mg/g of dry leaves	1.84 mg/g of dry fruit	-	[23]

^1^ = Essential oils, ^2^ = aerial parts, n.d. = not defined.

The non-polar extracts are the fractions with most of the interesting compounds, from a chemical and pharmacological point of view. Touihri et al. characterized the hexane extract of *E. elaterium* seeds, using Soxhlet extraction and obtaining fatty acids, linoleic acid (**98**) being the most abundant one (48.64%), tocopherols, with a high presence of γ-tocopherol (**110**) (44.23 mg/100 g of seed oil), and phytosterols, with β-sitosterol (**104**) being the most abundant phytosterol (396.25 mg/100 g of seed oil) [19]. Hexane extracts from aerial parts of this plant were studied by Molavi et al., who found a very high content of n-hentriacontane (**37**) (73.97% of the volatile components) [15]. Many studies focused on essential oils extracted from different plant tissues. The analysis of Jebara et al. focused on essential oils from aerial parts of *E. elaterium*, resulting in 31 different polar and non-polar compounds identified and quantified, and showing that hinesol (**18**), benzaldehyde (**1**) and β-ionone (**19**) were the main components of the essential oils (17.2%, 12.3% and 7.8%) [2]. Moreover, essential oils were extracted from plant leaves and fruits by Razavi and Nejad-Ebrahimi, who found a relatively high quantity of anethol (**48**) (31.6%), in fruits, and of octyloctanoate (**63**) and 3-(6,6-Dimethyl-5-oxohept-2-enyl)-cyclohexanone (**50**) (30% and 20.4%, respectively), in leaves [16].

The polar extracts of *E. elaterium* tissues are, on the other hand, less studied, compared to the non-polar component. Akinci and Losel characterized the soluble sugar content of the leaves from different *Cucurbitaceae*, including *E. elaterium*, and the analysis highlighted a high content of glucose (**114**) and fructose (**113**) (34% and 32% of the total sugar composition, respectively) [21].

## 3. Cucurbitacins: Chemical Structure

**Figure 2:**
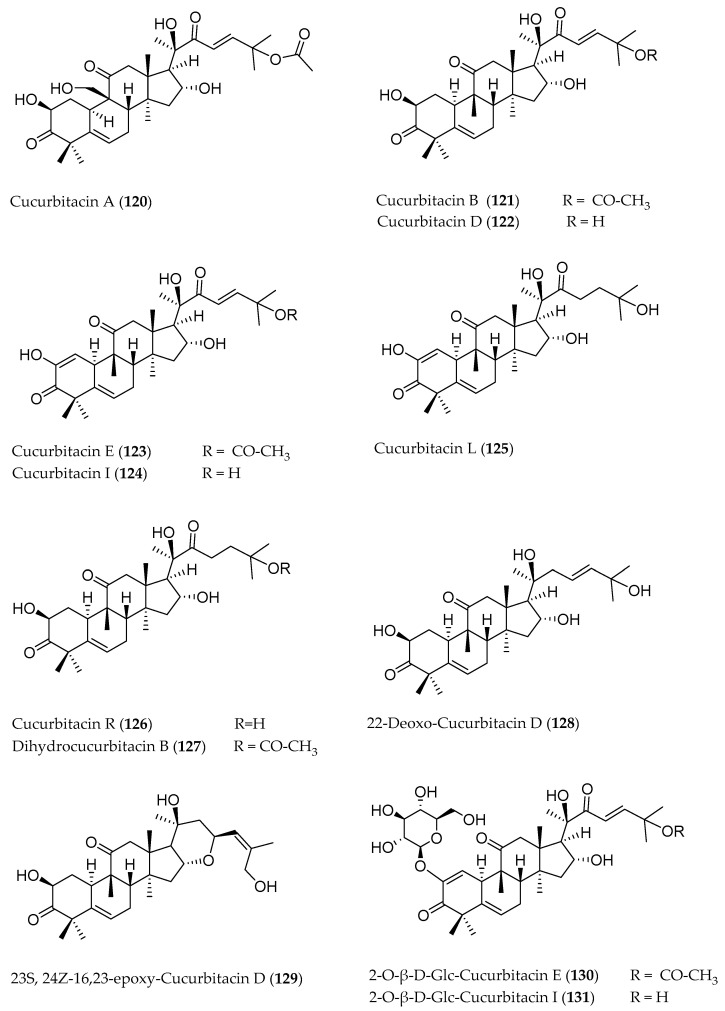
Chemical structure of cucurbitacins isolated from *Ecballium elaterium* leaves and fruits.

Cucurbitacins are a class of tetracyclic triterpenes produced from plants belonging to the *Cucurbitaceae* family and thus isolated from *E. elaterium* (**120**–**131**, Figure 2). They are usually found in plants as glycosides, hydrolyzed by the enzyme elaterase during the extractive procedures, and releasing the aglycone part [9]. *E. elaterium* is the plant from which the first cucurbitacin, named α-elaterin, was isolated back in 1831. This compound has been later been renamed as cucurbitacin E (**123**). The main function of these secondary metabolites is to protect the plant against external attacks of herbivores, thanks to their strong bitter taste. The basic skeleton of cucurbitacins is a core structure of cucurbitane (19-(10→9β)-abeo-10α-lanost-5-ene), which is then oxygenated and substituted with acetyl groups affording the different cucurbitacins (Figure 2). The main differences among the chemical structures are related to the double bonds at C-1 and C-23, and the presence of a hydroxyl or acetoxyl group at C-25. Other differences concern the glycosilation at C-2 and the closure of a further ring (ring E).

These compounds are extracted by using apolar aprotic solvents, e.g., chloroform, dichloromethane, petroleum ether, benzene, and ethyl acetate, but are also soluble in protic polar solvents such as methanol and ethanol. Strucure elucidation is obtained by spectroscopic methods, including MS, ^1^H-NMR, and ^13^C-NMR. Over the years, the research on cucurbitacins produced a wide literature on their extraction, which included different methods and involved the use of various solvents. Cucurbitacins are moderately polar compounds, which are soluble in organic solvents. They have been reported in the plant fruit. The aglycone is poorly soluble in water and highly soluble in chloroform [9]. The most-used solvents for the extraction of *E. elaterium* fruits, as shown in Table 2, were chloroform and dichloromethane, but also methanol, ethanol and petroleum ether, by using Soxhlet extraction, or solvent maceration of the dried fruit powder or fruit juice, under stirring.

The purification steps were usually performed by silica gel chromatography using the low polarity of these molecules to carry out multiple purification steps. In more recent works, the use of HPLC coupled with a UV detector is more commonly adopted, using acetonitrile, methanol and water as mobile phases, and coupled with UV as the detector.

Further information about the chemical structures of cucurbitacin were obtained by using spectroscopic techniques, including ^1^H-NMR, ^13^C-NMR and MS (Table 2). Furthermore, Sturm and Stuppner developed a method to simultaneously analyze the cucurbitacins present as aglycones and the glycosylated ones, using a precise HPLC-MS method [24]. The two glycosylated cucurbitacins analyzed in the work of El Sayed and Badr were extracted from the whole plant, using a 90% ethyl alcohol solution, purified by silica gel chromatography, and structurally elucidated through ^1^H-NMR [6].

In the studies that we considered, after the identification of the cucurbitacins, the authors rarely proceeded with quantification. The purification, identification and quantification processes are long and tedious, and the cucurbitacins are usually purified after multiple fractionation steps. The only studies that quantified cucurbitacins are the ones by Agil et al. [25], Yesilada et al. [26], Tosun and Baysar [27], Seger et al. [28], and El Sayed and Badr [6], as shown in Table 2.

**Table 2:** List of cucurbitacins found in *Ecballium elaterium* extracts and their correlated biological activity.

Name	Chemical Formula	Molecular Mass (g/mol)	Plant Part	Extraction Solvent	Method	Quantity	Biolological Activity	Ref
Cucurbitacin A (**120**)	C_32_H_46_O_9_	540.70	Fruit	Hexane	HPLC-DAD-ESI-MS	-	-	[17]
Cucurbitacin B (**121**)	C_32_H_46_O_8_	558.70	Fruit	Petroleum ether	Silica gel chromatography,IR, ^1^H-NMR, ^13^C-NMR	2.56% of the fruit juice	Anti-hepatotoxic effect in mice	[25]
			-	-	-	n.a.	Antimicrobial activity against *S. aureus*, antiviral activity against HSV-1	[8]
			Fruit	Dichloromethane	HPLC-MS, ^1^H-NMR, ^13^C-NMR	n.a.	-	[28]
			Leaves	Methanol	LC-ESI-MS	n.a.	Cytotoxic effect, inhibition of human glioma cell adhesion	[29]
			Fruit	Fruit juice	Preparative TLC ^1^H-NMR	2.57 mg crude/g dry juice	Anti-inflammatory activity induced by cucurbitacin B in mice	[26]
Dihydro-Cucurbitacin B (**127**)	C_32_H_48_O_8_	560.72	Fruit	Hexane	HPLC-DAD-ESI-MS	-	-	[29]
Cucurbitacin D (**122**)	C_30_H_44_O_7_	516.67	Fruit	Methanol, ethanol	HPLC-ESI-MS	n.a.	Decrease bilirubin level in human plasma	[5]
			-	-	-	n.a.	Inhibition of lung cancer cell proliferation	[30]
			Fruit	Methanol	Column chromatography^1^H-NMR	n.a.	Cytotoxic effect on gastric cancer cells	[31]
			Fruit	Dichloromethane	HPLC-DAD-MS, ^1^H-NMR, ^13^C-NMR	n.a.	-	[32]
			Fruit	Dichloromethane	HPLC-MS, ^1^H-NMR, ^13^C-NMR	6 mg	-	[28]
			Fruit	Water	HPLC-UV, ^1^H-NMR, LC-ESI-MS	86.4 µg/g of dried residue	-	[27]
			Fruit	Hexane	HPLC	n.a.	Cytotoxic, apoptotic, and anti-migration effects against hepatocellular carcinoma	[33]
22-Deoxo-Cucurbitacin D (**128**)	C_30_H_46_O_6_	502.33	Fruit	Dichloromethane	HPLC-MS, ^1^H-NMR, ^13^C-NMR	37 mg (mixture with Cuc. R)	-	[28]
			Fruit	Dichloromethane	HPLC-DAD-MS, ^1^H-NMR, ^13^C-NMR	n.a.	-	[32]
(23*S*, 24*Z*)-16,23-Epoxy Cucurbitacin D (**129**)	C_30_H_44_O_6_	500.67	Fruit	Dichloromethane	HPLC-MS, ^1^H-NMR, ^13^C-NMR	3 mg	-	[28]
Cucurbitacin E (**123**)	C_32_H_44_O_8_	556.69	Fruit	Chloroform	HPLC-DAD-UV	n.a.	Cytotoxic effect on Parkinson’s disease cells	[34]
			-	-	-	n.a.	Cytotoxic effect, immunostimulant effect	[35]
			-	-	-	n.a.	Cytotoxic effect on breast carcinoma cancer cells, melanoma and prostate adenocarcinoma	[36]
			Fruit	-	-	n.a.	Cytotoxic effect on ovarian cancer cells	[4]
			Fruit	Methanol	HPLC-UV	n.a.	-	[37]
			Fruit	Methanol	Column chromatography^1^H-NMR	n.a.	Cytotoxic effect on gastric cancer cells	[31]
			Fruit	Dichloromethane	HPLC-MS, ^1^H-NMR, ^13^C-NMR	n.a.	-	[28]
			Fruit	Dichloromethane	HPLC-DAD-MS, ^1^H-NMR, ^13^C-NMR	n.a.	-	[32]
			Fruit	Hexane	HPLC	n.a.	Cytotoxic, apoptotic, and anti-migration effects against hepatocellular carcinoma	[33]
2-*O*-β-d-Glucopyranosyl Cucurbitacin E (**130**)	C_38_H_54_O_13_	718.36	Whole plant	90% Ethyl alcohol	Silica gel chromatography,^1^H-NMR	46.67 mg/kg fresh plant	-	[6]
Cucurbitacin I (**124**)	C_30_H_42_O_7_	514.65	Fruit	Methanol, ethanol	HPLC-ESI-MS	n.a.	-	[5]
			Fruit	Chloroform	Column chromatography^1^H-NMR	n.a.	Cytotoxic effect on gastric cancer cells	[31]
			Fruit	Dichloromethane	HPLC-MS, ^1^H-NMR, ^13^C-NMR	n.a.	-	[28]
			Fruit	Dichloromethane	HPLC-DAD-MS, ^1^H-NMR, ^13^C-NMR	n.a.	-	[32]
			Fruit	Water	HPLC-UV, ^1^H-NMR, LC-ESI-MS	61 µg/g of dried residue	-	[27]
			Fruit	Hexane	HPLC-UV, ^1^H-NMR, ^13^C-NMR	n.a.	Anti-cancer effect against hepatocellular cancer cells	[38]
			Fruit	Hexane	HPLC	n.a.	Cytotoxic, apoptotic, and anti-migration effects against hepatocellular cancer cells	[33]
			Fruit	Chloroform	HPLC-UV	n.a.	Anti-cancer effects on breast cancer cells	[39]
2-*O*-β-d-Glucopyranosyl Cucurbitacin I (**131**)	C_36_H_52_O_12_	676.35	Whole plant	90 % Ethyl alcohol	Silica gel chromatography,^1^H-NMR	34.67 mg/kg fresh plant	-	[6]
Cucurbitacin L (**125**)	C_30_H_44_O_7_	516.67	Fruit	Dichloromethane	HPLC-MS, ^1^H-NMR, ^13^C-NMR	n.a.	-	[28]
Cucurbitacin R (**126**)	C_30_H_46_O_7_	518.68	Fruit	Dichloromethane	HPLC-MS, ^1^H-NMR, ^13^C-NMR	37 mg (mixture with 22-Deoxo Cuc. D)	-	[28]

n.a. = not available, DAD = Diode Array Detector; ESI = Electron Spray Ionization; HPLC = High-performance Liquid Chromatography; HSV = Herpes Simplex Virus; IR = Infrared; NMR = Nuclear Magnetic Resonance; MS TLC = Thin-Layer Chromatography, UV = Ultraviolet.

## 4. Cucurbitacins: Biological Activity

**Figure 3:**
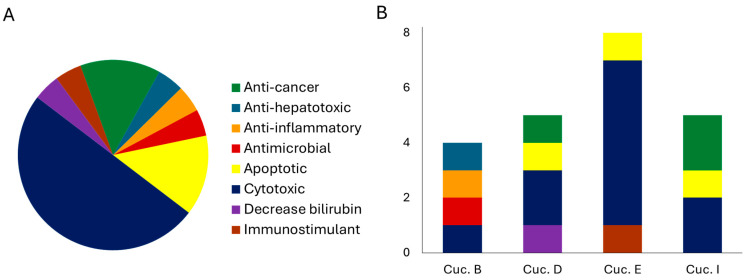
Biological activity exhibited by *E. elaterium* cucurbitacins (**A**) and the distribution of these activities within the single metabolite tested (**B**).

Investigations on the potential biological activity of cucurbitacin has resulted in the identification of biological properties (Table 2). Figure 3 shows the number of studies on the biological activity (A) and the distribution of these activities within the single metabolite tested (B). Interestigly, cyctotoxic and apoptotic activities were found for cucurbitacin D (**122**), E (**123**) and I (**124**), while anticancer activity was observed for D (**122**) and I (**124**). Cucurbitacin D (**122**) decreased bilirubin, while E (**123**) was shown to be an immunostimulant. Studies on cucurbitacin B (**121**) showed cytotoxic, anti-hepatotoxic, antimicrobial and anti-inflammatory activity (Figure 3B).

However, it has been found that the oral, subcutaneous, intraperitoneal and intravenous supply of pure cucurbitacin in various animals produce severe toxicity. The adverse effects resulting from pure cucurbitacin administration can vary, from general disorders like accelerated pulse, dyspnea, anorexia, diarrhea, and irritation of mucosa, to severe conditions like convulsions, asphyxia, and accumulation of fluids in organs, with the consequent organ failure and death. The most toxic cucurbitacins are cucurbitacins D (**122**) and I (**124**), with an LD_50_ (Lethal Dose 50) value of 5 mg/kg of body weight in mice. Both cucurbitacins have an unsaturated side chain and a free hydroxyl group, differing in a double bond at C-1 [9].

Going into detail in the tested biological activities, cucurbitacin B (**121**), extracted from *E. elaterium* fruits, was found to possess anti-hepatotoxic effects in mice, by Agil et al. [25]. Later on, Hassan et al. performed antimicrobial and antiviral tests of pure cucurbitacins B against *Staphylococcus aureus* and against the virus HSV-1, and confirmed the antimicrobial activity of this molecule [8]. The group of Yesilada et al. tested cucurbitacin B (**121**) in mice to assess the anti-inflammatory activity of this principle, finding an activity threshold of 50 mg/kg of mouse body weight [26]. Cucurbitacin D (**122**) was found to reduce the bilirubin level in human plasma by Greige-Gerges et al. [5], and was found to inhibit the proliferation of gastric cancer cells in the work of Jacquot et al. [30]. Moreover, Üremiş et al. isolated and tested cucurbitacin I (**124**) against hepatocellular cancer cells, and demonstrated the anti-cancer effect of the substance [38]. This finding adds up to the activity against breast cancer cells reported by Yılmaz and Deniz for cucurbitacin I (**124**) [40]

The most observed effect of cucurbitacins was the cytotoxic effect. Touihri-Barakati et al. found that cucurbitacin B (**121**) exerted cytotoxicity against human glioma cells, reducing their adhesive ability, as well [29]. Cucurbitacin D (**122**) cytotoxicity was tested against gastric cancer cells from Jafargholizadeh et al. [31] and against hepatocellular carcinoma by Üremiş et al. [33]. Additionally, cucurbitacin E (**123**) exhibited cytotoxic activity in many works, against Parkinson’s disease cells, against ovarian cancer cells, breast carcinoma cells, melanoma, prostate adenocarcinoma and hepatocellular carcinoma [4,31,33,34,36]. Finally, some works highlighted cucurbitacin I (**124**) cytotoxic activity against gastric cancer cells, hepatocellular cancer cells and breast cancer cells [31,33,39].

## 5. Biological Activity of Different Plant Tissues

*E. elaterium* has been used for more than 2000 years as a medicinal plant to treat several conditions such as rheumatism, jaundice, sinusitis, fever, liver disorders, constipation, hypertension, and cirrhosis [4,5,6,41]. Thus, starting from these traditional uses, many articles have tested the extracts from different parts of the plant and documented various biological activities. Table 3 reports all the biological and pharmacological studies available in the literature, with particular attention on the tested extract, the observed effect and target organism, and the tested concentration.

Antibacterial and antimicrobial activities were demonstrated for different extracts against *Klebsiella pneumoniae*, *Salmonella typhi*, *Staphylococcus aureus*, *Candida albicans*, *Bacillus subtilis*, *Salmonella enteritidis* in many articles that focused on chloroform, hexane, ethyl acetate, butanol, ethanol, aqueous and methanol fruit extracts [42,43,44,45]. The reason why these extracts can exert such activity is still under investigation, but Elkhateeb et al. [43] showed that the *E. elaterium* nanoparticles used in their study could break the cellular membrane of *S. typhi*, while Felhi et al. [45] concluded that the methanol extract they used was bacteriostatic against Gram-positive bacteria and bactericidal against Gram-negative bacteria, due to the difference in the membrane composition.

Several studies explored the anti-inflammatory activity against different types of diseases, suggesting that the content of cucurbitacins and the different polyphenols in *E. elaterium* parts could have a protective role against inflammation. Again, the extracts that exhibited anti-inflammatory activity were the fruit extracts and the fruit juice extracts, probably because of the cucurbitacin content, as shown in the article of Yesilada et al., which tested the extracts against mice, and isolated cucurbitacin B (**121**), correlating the anti-inflammatory activity with this metabolite [26]. Demir et al. [41] demonstrated the efficacy of fruit ethanolic extract against sepsis-induced lung injury, while Heysieattalab and Sadeghi [46] used fruit juice to reduce neuroinflammation in rats. Moreover, ethanol and methanol fruit extracts were used against inflammation of nasal mucosa and inflammation due to carrageenan-induced paw edema in rats [47]. An anti-inflammatory activity of *E. elaterium* fruit ethanol extract against sepsis-associated encephalopathy and fruit juice against liver fibrosis have been investigated by the groups of Arslan et al. [48] and Ghanim et al. [49], finding the fruit extracts effective in both cases.

**Table 3:** Biological activity of *Ecballium elaterium* extracts from the different plant tissues.

Activity	Material Tested	Plant Part	Disease	Observed Effect and Target Organism	Quantity	Ref.
Antibacterial	Hexane, chloroform, ethyl acetate, butanol, ethanol extracts	Fruit	-	Antibacterial effect against 10 *K. pneumonia* strains	MIC: 32–64 µg/mL	[42]
Anti-fibrotic	80% ethanol extract	Fruit	Fibrosis	Reduced fibrosis, wound healing in rats	5 mg/kg of body weight	[50]
Anti-inflammatory	Aqueous extract	Fruit	Rhinosinusitis	Anti-inflammatory activity in white rabbits	-	[51]
	80% ethanolic extract	Fruit	Sepsis-induced lung injury	Anti-inflammatory effect against sepsis-induced lung injury in rats	2.5 mg/kg of body weight	[41]
	Fruit juice	Fruit	Neuroinflammation	Anti-inflammatory activity in rats	10.9 µg/kg body weight 21.8 µg/kg body weight	[46]
	Fruit juice, dichloromethane extract	Fruit	Increased vascular permeability induced by HOAc	Anti-inflammatory activity induced by cucurbitacin B in mice	50 mg/kg of body weight	[26]
	Fruit juice, dichloromethane extract	Fruit	Increased vascular permeability induced by HOAc	Anti-inflammatory activity induced by cucurbitacin B in mice	50 mg/kg of body weight	[26]
	Fruit juice, ethanol extract	Fruit	Inflammation of the nasal mucosa	Anti-inflammatory activity in rats	100 mg/mL, 200 mg/mL and pure juice	[47]
	Methanol extract	Fruit	Carrageenan-induced hind-paw edema	Anti-inflammatory activity in rats	75 mg/kg	[45]
	80% ethanol extract	Fruit	Sepsis-associated encephalopathy	Anti-inflammatory activity in rats	2.5 mg/kg	[48]
	Fruit juice	Fruit	Liver fibrosis	Anti-inflammatory activity and hepatoprotective effect in mice	100 mg/kg of body weight	[49]
Antimalarial	Hexane extract	Root	-	Antimalarial effect determined by beta-hematin test	MIC: 4.645 mg/mL	[52]
	Dichloromethane extract		-		MIC: 2.637 mg/mL	
	Methanol extract		-		MIC: 0.124 mg/mL	
	Dichloromethane extract	Seed	-		MIC: 1.338 mg/mL	
	Methanol extract		-		MIC: 0.458 mg/mL	
	Hexane extract	Fruit	-		MIC: 3.518 mg/mL	
	Dicloromethane extract		-		MIC: 3.641 mg/mL	
Antimicrobial	Aqueous extract	Fruit	-	Antimicrobial activity against *S. typhi*	MIC: 10 µg/mL	[43]
	80% ethanol extract	Fruit	-	Antimicrobial effect against *S. aureus* and *C. albicans*	MIC between 0.195 and 1.563 mg/mL for *S. aureus* strains and between 0.097 and 6.250 mg/mL for *C. albicans*	[44]
	Methanol extract	Fruit	-	Antimicrobial activity in *S. aureus*, *B. subtilis*, *K. pneumonia*, *S. enteritidis*	MIC: 6 mg/mL (*B. aureus*, *B. subtilis*), 12 mg/mL (*K. pneumonia*, *S. enteritidis*)	[45]
Antioxidant	90% ethanol extract	Fruit	-	Antioxidant activity	IC_50_: 3.57 mg/mL	[53]
	Methanol extract	Fruit	-	Antioxidant activity	156 μg/mL	[45]
Anti-tumours	Seed oil	Seed	Glioma	Reduced adhesion of glioma cell lines (U87 cell lines) to fibrinogen (IC_50_ = 9.2 µg /mL), fibronectin (IC_50_ = 34.1 µg /mL).	Adhesion to fibrinogen (IC_50_ = 9.2 µg/mL), to fibronectin (IC_50_ = 34.1 µg/mL)	[29]
Antiviral	Fruit juice	Fruit	-	Antiviral activity against BRV/ERU_2018 strain	-	[3]
Cytotoxic	Aqueous extract	Fruit	-	Cytotoxic actiovity against AGS and KYSE30 cancer cell lines	IC_50_: 2.5, 0.7, 0.7 μg/mLfor AGS cells after 24, 48, 72 h. IC_50_: 500, 150, 125 μg/mL after 24, 48, 72 h for KYSE30 cells	[54]
	80% ethanol extract	Seed	-	Cytotoxic activity against MA-104 cell line	LogIC_50_: 6.03	[3]
	90% ethanol extract	Fruit	-	Cytotoxic activity against cancerous cell lines	IC_50_: between 1.953 and 702 μg/mL	[53]
	Fruit juice	Fruit	-	Cytotoxic activity against MA-104 cell line	LogIC_50_: 4.8	[3]
	Fruit juice	Fruit	-	Cytotoxic and mutagenic effect against human blood cells	72 µL/L for 48 h	[55]
	Fruit juice	Fruit	-	Cytotoxic and genotoxic effect against *A. cepa* meristematic cells	10, 20 and 50 mL/L and pure juice	[56]
Intraperitoneal adhesion reduction	80% ethanol extract	Fruit	Postoperative peritoneal adhesion	Reduction of postoperative intraperitoneal adhesions in rats	2.5 mg/kg of body weight	[57]
Pro-apoptotic	Fruit juice	Fruit	CCl_4_-induced hepatotoxicity	Pro-apoptotic effect in rats	0.7 mg/kg of body weight	[58]

MIC = Minimum Inhibitory Concentration, IC_50_ = Half Maximal Inhibitory Concentration, BRV/ERU = Bovine Rotavirus strain.

Cytotoxic activity was studied both in vivo and in vitro. Fruit juice was cytotoxic against monkey kidney cells, human blood cells and *A. cepa* meristematic cells, according to the results of Aksoy et al. [3], Rencüzogullari et al. [55], and Çelik and Aslantürk [56]. Furthermore, aqueous and ethanolic extracts were tested against other type of cancer cell lines, demonstrating their cytotoxic activity [3,53,54].

Other works from different research groups showed that fruits, seeds and roots of *E. elaterium* are effective against other conditions. İbiloglu et al. showed that an amount of 5 mg/kg of ethanol fruit extract can reduce fibrosis and enhance wound healing in rats [50]. Asgharian et al. tested different extracts from roots, seeds and fruits, and found an antimalarial effect of all the fractions, with an MIC ranging from 0.124 mg/mL to 4.645 mg/mL, suggesting that the phenolic content of the extracts could be correlated with the antimalarial effect [52]. Touihri-Barakati et al. studied the anti-tumour effect of *E. elaterium* seed oil against glioma cells: the application of seed oil decreased the adhesion of the cells to fibrinogen and to fibronectin, reducing the possible metastasis, with the fatty acids such as linoleic acid (**98**), oleic acid (**100**), palmitic acid (**101**) and punicic acid being indicated as possible responsible of such results [29]. Anti-viral activity against a bovine rotavirus (BRV/ERU) was obtained after using fruit juice on the target virus [3]. Fruit juice also exerted a pro-apoptotic activity in the work of Naggar et al., when the juice was administrated to rats with a dose of 0.7 mg/kg of rat weight, suggesting that cucurbitacin B, as shown in Table 3, could be the molecule that was exerting the pro-apoptotic activity. [58] On the other hand, ethanolic extract of fruit in rats was able to reduce the postoperative intraperitoneal adhesions, formations that can lead to abdominal pain and intestinal obstruction [57].

## 6. Conclusions

The biological importance of *E. elaterium*, which explain its use as a remedy in traditional medicine, is mainly due to the cucurbitacin content, and only in a minor extent to other chemical constituents found in the extracts from different plant parts. In this review, we brought to attention the chemical, physiological, and biological characteristics of this plant, which has not been very much studied in the past years. Future studies should investigate and clarify the molecules responsible for some of the tested biological activities, still not clarified today, and determine whether or not such in vivo and in vitro activities are ascribable to the isolated cucurbitacins. Also, some efforts could aim to fill the lack of information and knowledge about the glycosylated cucurbitacins and, eventually, clarify their positive and negative effects.

## Data Availability

Data will be provided by the authors, on request.
